# Functions of Lipids for Enhancement of Oral Bioavailability of Poorly Water-Soluble Drugs

**DOI:** 10.3797/scipharm.1105-09

**Published:** 2011-08-07

**Authors:** Basavaraj K. Nanjwade, Didhija J. Patel, Ritesh A. Udhani, Fakirappa V. Manvi

**Affiliations:** Department of Pharmaceutics, KLE University College of Pharmacy, Nehrunagar, 590010, Belgaum, India

**Keywords:** Lipids, Lipid digestion, Mean emulsion droplet diameter, Self-emulsifying drug delivery system, Isotropic lipid solutions

## Abstract

Lipid-based formulations encompass a diverse group of formulations with very different physical appearance, ranging from simple triglyceride vehicles to more sophisticated formulations such as self-emulsifying drug delivery systems (SEDDS). Lipid-based drug delivery systems may contain a broad range of oils, surfactants, and co-solvents. They represent one of the most popular approaches to overcome the absorption barriers and to improve the bioavailability of poorly water-soluble drugs. Diversity and versatility of pharmaceutical grade lipid excipients and drug formulations as well as their compatibility with liquid, semi-solid and solid dosage forms make lipid systems most complex. Digestion of triglyceride lipids, physicochemical characteristics and solubilisation of lipid digestion products as well as intestinal permeability are some of the variable parameters of such formulations. Furthermore, among the factors affecting the bioavailability of the drug from lipid-based formulations are the digestion of lipid, the mean emulsion droplet diameter, the lipophilicity of the drug and the type of lipids. The solubility of the Active Pharmaceutical Ingredient in the Lipid System, the desorption/sorption isotherm and the digestibility of lipid vehicle are important issues to be considered for formulations of isotropic lipid formulations. This review also describes the fate of lipid formulations in the gut and the factors influencing the bioavailability from lipid-based formulations. Novel formulation systems and currently marketed products conclude this review.

## Introduction

Oral lipid-based products entered the market in 1981 and currently account for approximately 3% of commercially available oral formulations. Lipids are ubiquitously distributed compounds that play fundamental roles in the architecture and functionality of all living cells and therefore are most commonly studied as components of foodstuff and important energy sources in enteral nutrition [1]. The total daily dose of active pharmaceutical excipient (API) administered by these formulations ranges from 0.25 μg to 2000mg. For capsules, the amount of active drug substance per dosage unit ranges from 0.25 μg to 500 mg, and for oral solutions from 1 μg/ml to 100 mg/ml. The total amount of lipid administered in a single capsule ranges from 0.5 to 5 g, but can be 0.1 ml up to 20 ml for oral solutions [2]. Lipid-based formulations range in complexity from simple, one-excipient solutions based on e.g. sesame or corn oil to multi-excipient, self-emulsifying drug delivering systems (SEDDS). The excipients in lipid-based formulations include water-insoluble triglycerides (e.g., corn oil, olive oil, peanut oil, sesame oil, soyabean oil, rapeseed oil, hydrogenated vegetable oil, hydrogenated soybean oil and medium chain triglycerides of coconut oil and palm seed oil), organic liquids/semisolids (e.g., beeswax, DL-α-tocopherol, oleic acid, medium chain mono and diglycerides, propylene glycol ester of fatty acids), nonionic surfactants (e.g., cremophor EL, cremophor RH 40, cremophor RH 60, d-α-tocopherol propylene glycol 1000 succinate, glyceryl monooleate, polysorbate 20, polysorbate 80, Sorbitan monolaurate, Labrafil M-1944CS, Labrafil^®^ M-2125CS, and Labrasol^®^), a phospholipid (phosphatidylcholine), and water-miscible organic solvents (polyethylene glycol 400, ethanol, propylene glycol, glycerin).

There is increasing demand to develop suitable drug carrier systems in order to control, localize and improve drug delivery. At that, many natural and synthetic lipids are aimed to improve oral bioavailability of poorly water-soluble drugs. When administered in hydrophilic form of solid formulations, these compounds exhibit low bioavailability as their absorption can be dissolution- and capacity-limited due to poor solubility.

Lipid formulations can reduce the inherent limitation of slow and incomplete dissolution of poorly water-soluble drugs and facilitate formation of solubilised phases from which absorption may occur [3]. The solubilised phases most likely arise from intralaminal processing after lipid absorption. The co-administration of lipids with drugs can also impact their absorption pathway [4]. Although most orally administered compounds gain access to the systemic circulation via the portal vein, some highly lipophilic drugs are transported directly to the systemic circulation via intestinal lymphatics, which improves oral bioavailability of API.

Lipids open a wide array of different formulations for oral administration because they can be manufactured as solutions, suspensions, emulsions, self-emulsifying systems and micro-emulsions [4]. Lipids offer the potential for enhancing drug absorption and oral bioavailability. This review is aimed to provide an overview, how lipid-based formulations can improve drug absorption and oral bioavailability.

## Impact of the GI-tract on Lipids and Lipid-Based Formulations

The GI-tract basically influences the performance of lipid-based formulations as triglycerides and their derivatives are quantitatively hydrolyzed prior to absorption. After a meal, the presence of lipid digestion products induces secretion of pancreatic and biliary fluids which alter the luminal environment of the small intestine [5]. The lipid digestion products, being better water-soluble than the parent triglyceride, are solubilized within bile salt mixed micelles and for their subsequently delivered to absorptive cells of the GI-tract. The chemistry and nature of the interaction of lipid digestion products with the aqueous contents of the GI-tract change as a function of digestion and solubilization.

### Digestion of triglycerides

Intestinal absorption of the products of lipid digestion is the result of three sequential processes, which involve (i) dispersion of fat globules (ingested lipids) to yield a coarse emulsion of high surface area, (ii) enzymatic hydrolysis of the fatty acid glyceryl esters (primarily triglyceride lipids) at the oil/water interface and (iii) dispersion of the lipid digestion products into an absorbable form [6]. Salivary glands secrete lingual lipase and gastric mucosa secretes gastric lipase, which initiate triglyceride (TG) hydrolysis to the corresponding diglyceride (DG) and fatty acid (FA) within the stomach. The pH-optimum ranges for lingual and gastric lipase from 3 to 6, and medium chain triglycerides are hydrolyzed at a faster rate than long chain triglycerides [7].

TG are preferably hydrolysed by pancreatic lipase, an interfacial enzyme, which preferentially acts at the surface of emulsified TG droplets to quantitatively convert TG into the corresponding 2-monoglyceride (MG) and two fatty acids (FA). Optimum activity is observed when oleate is the fatty acid, and it decreases towards shorter chain triacylglycerols and soluble carboxyl esters substrates. Bile salts at concentrations usually present in the duodenum inhibit binding of pancreatic lipase at the oil-water interface.

**Fig. 1:**
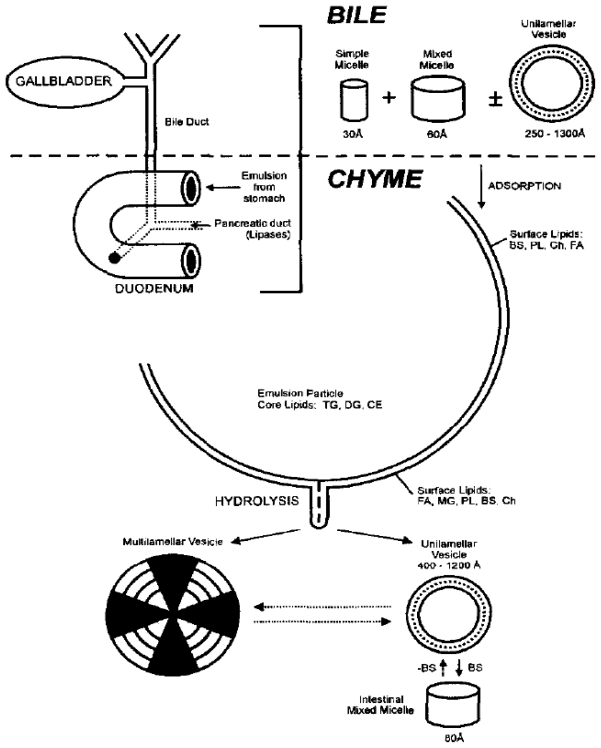
Model for sequential steps involved in lipid digestion

Further classes of biliary-derived lipids are the phospholipids (e.g., phosphatidylcholine, PC) playing an important role as solubilisers for lipid digestion products. Prior to absorption, PC is hydrolyzed by phospholipase A_2_ to lysophophatidylcholine (lyso-PC), with the majority of PC present in the intestine secreted in bile (10–20 g/day) with only modest dietary influence. Model for the sequential steps involved in lipid digestion is given in Fig. 1.

### Physical chemistry, absorption and solubilisation of lipid digestion products

Bile plays a key role in solubilisation of lipid digestion products and poorly water-soluble drugs [4]. The presence of lipid digestion products as well as cholecystokinin-mediated contraction of the gall bladder and relaxation of the sphincter of Oddi leads to expulsion of bile, with peak flow occurring approximately 30 min after ingestion of a meal.

Typical concentrations of bile salts in the fasted intestine are 4–6 mM compared with post-prandial concentrations of 10–20 mM [8]. The inclusion of the lipidic components decreases the critical micelle concentration (CMC) and an increase the size and solubilisation capacity of the micelles, e.g., the inclusion of lecithin and MG decreases the CMC of mixed micellar systems to values less than 1 mM. Therefore, it is likely that the CMC of mixed bile salt systems present in the intestine is surpassed even in the fasted state. Although the specific mechanisms of absorption of the lipid digestion products have not been elucidated yet, the common role of the intestinal mixed micellar phase to solubilise these poorly water-soluble compounds and to provide a concentration gradient for absorption of lipids and presumably of drugs solubilised in this phase is generally accepted. Micelles are not absorbed intact, and lipids are suggested to be absorbed from a monomolecular intermicellar phase [9–11]. The dissociation of monomolecular lipids from the mixed micellar phase prior to absorption may be stimulated by a microclimate of lower pH at the intestinal absorptive site [12, 13].

### Lipids and GI-transit profiles

The products of lipid digestion delay gastric emptying, and much pharmaceutical interest has focused on the relationship between gastric emptying, food ingestion, and the transit behavior of non-disintegrating controlled release dosage forms. Although there is little information as to how small pharmaceutically relevant volumes of lipid impact on transit profiles, it is likely that any effect would not significantly impact the bioavailability of the co-administered drug [4].

### Lipids and intestinal permeability

Constituents of the mixed micellar phase can modify the intestinal permeability of poorly water-soluble compounds via three mechanisms. Firstly, the presence of lipid digestion products and bile salts may alter the intrinsic permeability of the intestinal membrane, leading to increased absorption via paracellular or transcellular routes. Secondly, solubilisation of lipophilic drugs within bile salt mixed micelles may facilitate diffusion through the aqueous diffusion layer, improving absorption. Thirdly, and conversely, drug solubilisation may decrease the intermicellar ‘free’ fraction of drug, which could potentially lead to a decrease in absorption. These divergent effects are likely to provoke sometimes contradictory reports about drug absorption after administration as solubilised surfactant-based systems [8].

## Factors Affecting Bioavailability of Lipid-Based Formulations

### Lipid Digestion

**Fig. 2:**
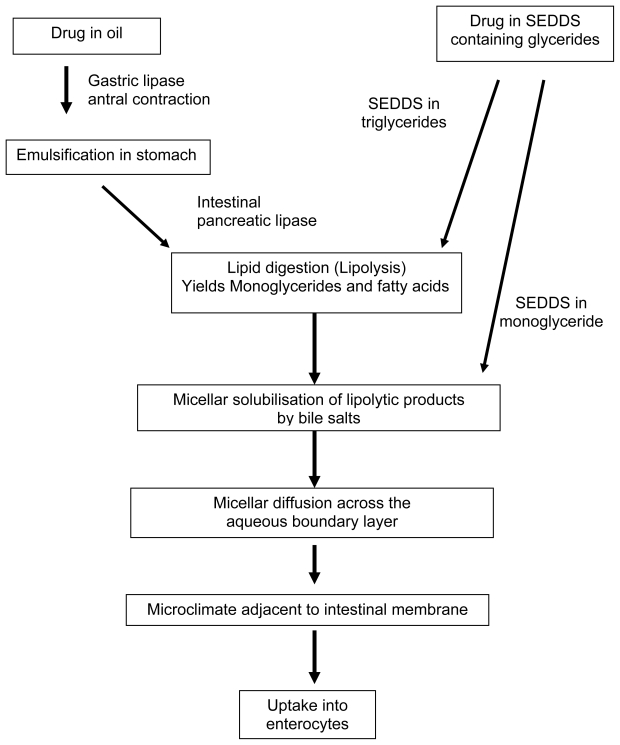
Schematic diagram of the fate of lipids in the gut.

If the drug possesses high affinity to the lipid vehicle, it can be assumed that the API moves apparently together with vehicle in the GI-tract, revealing that digestibility of lipid would be as important as gastric emptying rate of the same. Thus, careful selection of the lipid vehicle can control the absorption rate of drug. GI lipid digestion consists of three sequential steps: (i) the dispersion of fat globules to yield a fine emulsion, (ii) the enzymatic hydrolysis of fatty acid esters at the emulsion-water interface, and (iii) the desorption and dispersion of insoluble lipid products for subsequent absorption [14, see Fig. 2].

The API accumulated at the surface of the absorptive epithelium is taken up by enterocytes. Certain drugs and surfactants reduce the activity of efflux transporters in the GI-wall and, hence, increase the fraction of drug absorbed. Because of the interplay between P-gp and CYP3A4 activity, this mechanism may reduce intracellular metabolism as well. Non-digestible lipids, including mineral oils, sucrose polyesters and others, are not absorbed from the gut lumen. They remain in the gastrointestinal lumen, tend to retain the lipophilic drug within the oil, and thus, may limit the absorption of the drug. Consequently, lipids that are not affected by negative effects of surfactant such as medium chain monoglycerides, fatty acids, and monoesters of fatty acids are preferred as vehicles for lipid delivery systems.

### Mean emulsion droplet diameter

The mean emulsion droplet diameter is a parameter indicating the quality of self-emulsifying formulations. The droplet size of SEDDS upon dilution with aqueous media is primarily influenced by the type and concentration of emulsifier: The higher the concentration of emulsifier, the smaller the emulsion droplet and the faster the drug release. Two techniques are commonly used to determine the mean emulsion droplet diameter: low angle laser light diffraction is applied for emulsions with droplet sizes > 1μ and quasi-elastic light scattering for investigations of submicron dispersions. [15–17].

In addition, the mean emulsion droplet diameter seems to be a very critical factor for prediction of the *in-vivo* performance of undigested lipid-based formulations, such as long chain triglycerides in case of Cyclosporine (Neoral^®^ versus Sandimmune^®^) [18]. Nevertheless, in case of predigested lipids such as medium chain monoglycerides or propylene glycol monoester of C8–C10 fatty acids, the mean emulsion droplet diameter may not be crucial *in-vivo.*

### Lipophilicity of the API

Highly hydrophobic drugs (log P > 6) can be taken up into the lymphatic system by partitioning into chylomicrons in the mesentery vein [19] which has been demonstrated to be crucial for the absorption of the anti-malaria compound halofantrine [20, 21]. Furthermore, highly lipophilic retinoids are known to be transported in the intestinal lymph after oral administration.

### Type of lipids

The nature or type of lipids is important because digestible lipids may influence absorption in a manner differing from that of non-digestible lipids. Commonly used digestible lipid vehicles are listed in table 1. The lower the melting point of the fatty acid, the higher is the amount of drug absorbed.

### Drug release

Primarily, the characteristics of drug and lipid as well as their aqueous solubility are key factors that control drug release and absorption from lipid-based formulations [22]. Other issues to be considered are whether the drug is formulated in oil, SEDDS or emulsified form, the absorption pathway of the drug, the droplet size of the emulsion present in the intestine, the type of surfactants, the metabolic pathway of the lipids and the changes in gastric motility due to presence of lipids.

## Crucial Issues during Development of Isotropic Lipid Solutions

The physico-chemical properties of the API as well as the choice of lipids play a major role in designing isotropic solutions and will decide whether it will be a simple lipid solution, a SEDDS or a SMEDDS. Some of the physico-chemical parameters, which influence the design of isotropic solution, include:

### Solubility of API in the Lipid System

Solubility is one of the major factors that limit the use of lipid-based delivery systems. The amount and the solubility of the drug in the lipid vehicle are decisive whether an isotropic lipid formulation is formed or not. Factors that can impact solubility include:

#### Solubility Parameters

The solubility characteristics are useful as a predictive tool for the selection of a solvent or solvents for a particular application. Solubility parameters give a systematic estimate for the compatibility between two components. As a rule of thumb two substances are miscible when their Hildebrand solubility parameters are close to each other or when the difference in their solubility parameters is less than 2 MPa^1/2^. Checking the Hildebrand solubility parameters is an effective way to predict the formulation characteristics of a particular compound.

#### Hydrophilic-Lipophilic Balance (HLB)

The HLB is an empirical formula that is used to characterize surfactants and to select those appropriate for preparation of microemulsions [23, 24]. Non-ionic and ionic surfactants are often considered for pharmaceutical applications because they are less toxic [26] and less affected by pH and ionic strength changes. The HLB value of each lipid vehicle is calculated according to:

$$\text{HLB}=20\frac{1-\text{S}}{\text{A}}$$

where S is the saponification number of the ester and A is the acid number of the fatty acid [27]. The higher the HLB value of the surfactant, the broader is the range to obtain micro-emulsions. Emulsifiers with HLB > 10, which are commonly used in isotropic lipid-based solutions, are summarized in table 2. Co-emulsifiers with HLB values ranging from 4 to 6, which are commonly used in isotropic lipid-based solutions, are given in table 3.

#### Partition Coefficient

This factor describes the lipophilicity of a molecule corresponding to the partition coefficient of a compound between a lipophilic and a hydrophilic phase, usually 1-octanol and water [28]. The partition coefficient is the concentration of the drug in the organic layer divided by that in the aqueous one. Finally, log P is defined as the decadic logarithm of P.

Compounds with log P>4 (i.e., being more lipophilic) are likely to be dissolved in oils. Compounds with intermediate log P (log P<4) may require a mixture of hydrophilic surfactants (HLB 4–12) or water-soluble co-solvents to form a self-emulsifying system with maximum solubility. Interestingly, a compound with high log P cannot necessarily exhibit high solubility in oil, whilst another compound may be highly soluble in oil but showing a relatively low log P value [29].

#### Phase Diagrams of lipid formulations

**Fig. 3:**
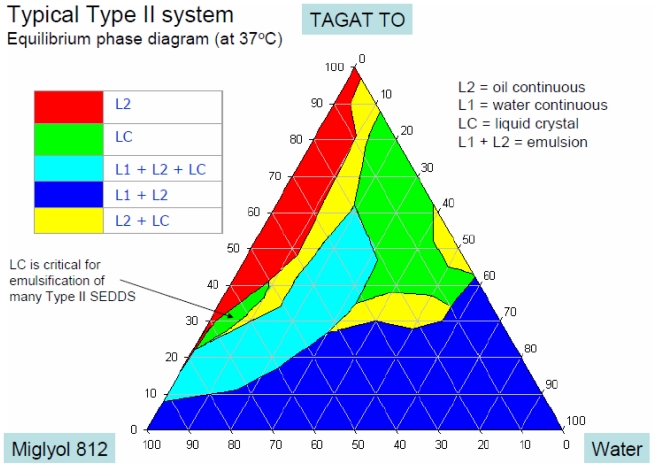
Schematic diagram of an Equilibrium phase for Type II SEDDS system (from Dumanli, I., Characterization of gelling phenomenon of a lipid-based formulation. Master Thesis, University of Rhode Island, 1998).

A phase diagram of the lipid formulation for a given API should be individually established by varying the ratio of drug, oil, and surfactant due to the impact of the physico-chemical properties of the drugs such as inherent polarity and surface activity. That way, different regions for good, intermediate, and poor self-emulsification are recognized. In addition, phase studies are performed by diluting a mixture of oil-surfactant sequentially with increasing amounts of water. After equilibrium, the phase type is identified using a crossed polarized viewer and an optical microscope (Fig. 3).

#### Drug Loading

It can affect long-term physical stability of the drug product. As a prerequisite, the saturation concentration of the API in the lipid-based formulation should be carefully established and drug loading has to be optimized in order to avoid potential gelling or drug crystallization under shear stress and/or during storage. Drastic changes in emulsifying properties as indicated by an increasing mean emulsion droplet diameter due coalescence and/or aggregation indicate product instability.

### Sorption/desorption (hygroscopicity) Isotherm

Hygroscopicity of the lipid may induce dehydration of the soft gelatin capsule and hard gelatin capsule shell, resulting in brittleness. Impact of temperature and moisture on the drug solubility characteristics must be investigated. Potential solute migration into the shell must be characterized during the formulation development, particularly when such a drug has good solubility in glycerin and sorbitol, which are commonly used plasticizers in soft gelatin capsules.

#### Shear effect

Prolonged shearing was shown to change the rheological behavior of an amorphous drug from Newtonian to pseudoplastic after dissolution in mono- and diglycerides [30]. Changes in the viscous modulus determined by oscillatory measurements indicate formation of structural elements due to presence of hydrogen bonds between drug and the vehicle. These interactions can also be detected by DSC and FT-IR. Moreover, the shear effect was shown to depend on drug concentration. Supersaturated drug solutions are more likely prone to gelation upon shear stress and/or precipitation upon the storage.

## Lipid Formulations And Oral Bioavailability

The simplest approach to evaluate absorption of drugs from lipid-based systems is to prepare a solution or suspension in lipids and do in-vivo assays (see table 4).

An early stimulus for the use of lipid vehicles for drug formulation was the observation of improved bioavailability when poorly water-soluble drugs were co-administered with food. A well-known example is the suspension of phenytoin in corn oil [31] which considerably improved oral bioavailability as compared to the aqueous suspension.

Abrams et al. studied the oral dose proportionality of a lipophilic steroid derivative, (5α,17β)-2-chloro-3-[(4-nitrophenoxy)imino]androstan-17-yl acetate (an *o*-aryl oxime of 2α-chlorodihydrotestosterone; nisterime acetate) when administered to rats as either solution in sesame oil or aqueous suspension [32]. The threefold higher bioavailability from sesame oil solution was attributed to improved solubilisation and dissolution in oil, which represent prerequisites for enhanced absorption.

Unfortunately, many poorly water-soluble drugs also exhibit limited solubility in pharmaceutically relevant lipids. A promising approach to improve lipid solubility of drugs with insufficient lipid solubility is the design of low melting, lipophilic prodrugs. For example, Yamoka et al. took account for the low equilibrium lipid solubility of phenytoin by synthesising a range of low melting prodrugs with enhanced lipid solubility [33].

The enhancement in oral bioavailability of a hydrophobic amine antimalarial, prepared as an oleic acid solution in a soft gelatin capsule, is typical for the situation where formulation of a poorly water soluble drug in lipid can provide effective oral delivery [34].

Oral administration as a solution in oleic acid was also effective in enhancing the absorption of cinnarizine. [35].

## Digestibility of the Lipid Vehicles

Lipids are typically classified according to their chemical structure, polarity, characteristics, and degree of interaction with water. Nevertheless, when assessing lipids as vehicles, their digestibility has to be considered. Non-digestible lipids such as mineral oil, e.g. liquid paraffin and sucrose polyesters, essentially remain unabsorbed in the intestinal lumen and can actually limit or even reduce drug absorption by retaining a considerable amount of co-administered drug. Digestive lipids comprise dietary lipids such as glycosides, fatty acids, phospholipids, cholesterol esters as well as various synthetic derivatives. The rate and extent of digestion, the colloidal phases formed and pharmacological effects of the digestion products represent potential factors influencing release of the API from the vehicle and its absorption. Table 5 lists selected studies where the effects of different lipids on drug absorption have been investigated.

Patton and Gilford demonstrated improved oral bioavailability of triamterene in rats when administered in 1ml lipid solution as compared to aqueous suspensions [36].

Myers and Stella [37] investigated the effects of non-digestible and digestible lipid vehicles on the oral bioavailability of penclomedine, a highly lipophilic, poorly water-soluble cytotoxic agent. The absolute bioavailability of penclomedine in the rat after administration in various lipids is higher than that of the aqueous suspension.

The dependency of cyclosporine absorption on bile secretion has also been evaluated after administration of a coarse lipid emulsion to liver transplant patients with a T-tube inserted in their common bile duct [38]. The absorption of cyclosporine was assessed before and after clamping the T-tube (i.e. with or without bile diversion) and the relative bioavailability of cyclosporine was 3–4 fold higher in the patients without bile diversion.

## Novel Lipid Formulations

The carrier systems that have been most extensively studied to control the release of the incorporated substances are:

- Liposomes
- Polymeric nano- and microparticles
- Solid lipid nanoparticles (SLNs)
- Self-emulsifying drug delivery systems (SEDDS)

Liposomes are spherical particles composed of one or more concentric phospholipid bilayers. This kind of structure makes it possible to incorporate lipophilic drugs into lipid bilayers as well as hydrophilic drugs into the aqueous compartment. Drug release from liposomes, stability, and pharmacokinetic profiles depend on composition, size, surface charge, and drug solubility. Liposome formulations of many different drugs show a significant increase in therapeutic activity compared with non-liposomal formulations. Liposomes are mostly biocompatible and biodegradable, but also face some disadvantages including low stability, low encapsulation efficiency, and difficult scale-up [38–42].

Polymeric nano- and microparticles are general terms that include spheres as well as capsules. Hydrophilic and lipophilic drugs can be incorporated or entrapped into these polymeric carriers, but reaching therapeutic levels is one the most crucial issues. These kinds of drug carrier systems have proved to be more stable than liposomes both *in-vivo* and during storage. Their main disadvantages are that preparation methods sometimes require organic solvents and that large-scale production is rather difficult. Moreover, it is sometimes crucial to find appropriate polymers that are confirmed to be nontoxic, biodegradable and nonimmunogenic [39–45]. Some synthetic polymer matrices have also been suspected to exert detrimental influence on peptides and proteins incorporated during the manufacturing process [45].

SLNs were developed in the early 1990s and represent promising drug carrier systems, especially in terms of mediating a sustained-release profile of the incorporated API. SLNs are in the submicron size range (50–1000 nm) and built from a lipidic matrix being solid at room temperature. They seem to represent an alternative drug carrier system to liposomes and polymeric nanoparticles. SLNs indeed combine several of their advantages while avoiding some of their disadvantages [46, 47]: (i) the lipids used are similar to physiological lipids so that toxicity is reduced. (ii) SLNs are physicochemically stable and can be produced easily on a large industrial scale. In addition, (iii) raw materials and production costs are relatively low. Their most important limitation is that the drugs incorporated into SLNs have to be sufficiently lipophilic to guarantee high entrapment efficiency. So far, SLNs have been studied for parenteral, oral, and topical administration [48–50].

Whereas the composition of solid lipid microspheres is equivalent to SLNs, their size is in the micrometre range. Given similar composition, these lipophilic particles may also be considered as physiologically compatible, physicochemically stable, and appropriate for up-scaled production. Only the difference in size between SLNs and solid lipid microparticles will decide on their application fields and administration.

The utility of solubilizing lipid based formulations to improve the gastro intestinal absorption of poorly water-soluble, hydrophobic drugs is well documented [1, 4]. It is suggested that improved absorption is predominantly due to the higher solubilisation rate representing a prerequisite for absorption in the GI-tract. Other mechanisms proposed include protection of the API inside the lipophilic vehicle from chemical and enzymatic degradation, which is localized in the aqueous environment, and promotion of lymphatic drug transport, which escapes hepatic first pass metabolism [19].

Use of lipid-based, self-emulsifying drug delivery systems (SEDDS) and self-micro-emulsifying drug delivery systems (SMEEDS) represent alternative formulations for improving oral absorption of poorly water-soluble drugs.

SEDDS and SMEDDS are easily manufactured and physically stable isotropic mixtures of oil, surfactants, co-surfactants and solubilised drug substance that are administered orally in soft or hard gelatin capsules. SMEDDS readily disperse in the GI-tract, where the motility in the stomach and intestine allow for emulsification [15]. Self-emulsifying properties are conferred by a proper selection of the lipid/surfactant pair as well as the optimum ratio of lipid and surfactant [15, 16]. In order to reach the optimum HLB value required for the emulsification, mixtures of different surfactants are in use [51].

The lipid droplets formed in self-emulsifying formulations may facilitate drug absorption directly, independent from the bile salt mediated mixed micelle transport system. The improved drug absorption provided by self-emulsifying formulations, however, depends on the maintenance of the drug in the solubilised state before it can be absorbed from the GI-tract.

## Marketed Lipid-Based Dosage Forms

Lipids are versatile tools for drug administration because they cover a wide array of different types of formulations, such as solutions, suspensions, emulsions, self-emulsifying systems, lipid particles, and microemulsions. The commercially available oral lipid-dosage forms are either filled in capsules or represent bulk oral solutions. Both soft and hard gelatine capsules are used for lipid-based formulations. Usually, liquid formulations are manufactured either as soft or hard gelatine capsules, whereas semisolid or solid formulations are filled into hard gelatine capsules. Parenteral formulations include emulsions, microemulsions, liposomes, lipid complexes, suspensions, and colloidal dispersions. The different kinds of formulations are generally divided into following categories:

- One-lipid excipient formulations
- Two-lipid excipients formulations
- Three-lipid excipients formulations
- Four-lipid excipients formulations
- Extended release formulations
- Microemulsions
- Oral suspensions s
- Oral solutions

### One-lipid excipients formulations

The simplest lipid-based formulations contain only one excipient such as oleic acid, α-tocopherol, corn oil, peanut oil, sesame oil, medium chain triglycerides, or medium chain mono- and diglycerides. All such oral formulations are marketed in soft gelatin capsules. Many of the over-the-counter sold products contain polyethylene glycol or medium chain triglycerides as the solubilizing excipients (see Table 6).

### Two-excipients formulations

The next level of complexity in lipid-based formulations is reached by those that contain two excipients. Some typical combinations are sesame oil with α-tocopherol, medium chain triglycerides with ethanol, and propylene glycol esters of fatty acids with glyceryl mono-oleate (see Table 7).

### Three-excipients formulations

Ascending the scale in complexity, lipid-based formulations containing mixtures of three excipients have to be mentioned. Typical examples of such combinations include: (i) TPGS, polyethylene glycol (PEG) 400, and PG; (ii) oleic acid, cremophor EL, and ethanol or PG; iii) polysorbate 20, PEG 400, and povidone; (iv) medium chain mono- and diglycerides, α-tocopherol, and povidone; and (v) medium chain triglycerides, glycol esters of fatty acids, and aspartic acids (see Table 8).

### Four-excipients formulations

The most complex lipid-based formulations currently marketed contain mixtures of four excipients such as (i) ethanol, cremophor EL, propylene glycol and mono/diglycerides of caprylic/capric acid; (ii) rapeseed oil, beeswax, hydrogenated soyabean, and partially hydrogenated plant oils. All the formulations are marketed in soft gelatin capsules (see Table 9).

### Extended release formulations

There are at least three commercially available extended release formulations that contain lipid excipients that are filled into hard gelatin capsules (see Table 10).

### Microemulsions

Microemulsions are thermodynamically stable, clear dispersions composed of a polar solvent, oil, surfactant, and co-surfactant. Microemulsions have demonstrated considerable potential for drug delivery due to their ability to solubilise highly hydrophobic drugs in both oral and intravenous administration (see Table 11).

### Oral solutions

Oral solutions are usually applied by pediatric patients or patients with inability to swallow a tablet or capsule. Some lipid containing oral solutions are relatively simple formulations, containing a single excipient, while others are complex mixtures of surfactants, solvents, flavour, sweeteners, and salts (see Table 12).

### Oral suspensions

Similar to oral solutions, oral suspensions are usually intended for pediatric patients or for patients who cannot swallow a tablet or capsule. The suspensions are supplied in two bottles, the one contains drug encapsulated into solid microcapsules together with polymers and other excipients, while the other bottle contains the diluent composed of medium chain triglycerides, sucrose, lecithin, water, and strawberry flavor. Prior to administration, the contents of the two bottles are mixed to form the suspension (see Table 13).

## Conclusion

Lipid-based formulations are an important tool for water-insoluble drugs as they offer the potential for enhancing drug absorption and oral bioavailability. The GI-tract basically influences the performance of lipid-based formulations. Especially the chemistry and nature of the interaction of lipid digestion products with the aqueous contents of the GI-tract changes according to digestion and solubilisation. Solid dosage forms of lipid formulations can improve stability, but may have lower drug loading and higher potential for drug crystallization. Thus, understanding and controlling phase transformations of the formulation are important.

## Figures and Tables

**Tab. 1:** Commonly used lipid vehicles for lipid-based formulations.

Classification	Lipophilic vehicles
Fatty acids	Oleic acid, Myristic acid, Caprylic acid, Capric acid
Ethyl esters	Ethyl Oleate
Triglycerides of long-chain fatty acids	Soybean Oil, Peanut Oil, Corn Oil
Triglycerides of medium-chain fatty acids	Miglyol^®^ 812, Captex^®^ 355, Labrafac^®^

**Tab. 2:** Commonly used emulsifiers in isotropic lipid based formulations.

Classification	Emulsifiers
PEGylated glycerides	PEG-8 glyceryl caprylate/caprate (Labrasol^®^) PEG-32 glyceryl laurate (Gelucire^®^ 44/14) PEG-32 glyceryl palmito stearate (Gelucire^®^ 50/13)
Polyoxyethylene sorbitan fatty acid esters	Polyoxyethylene 20 sorbitan monolaurate (Tween^®^ 20) Polyoxyethylene 20 sorbitan monostearate (Tween^®^ 60) Polyoxyethylene 20 sorbitan monooleate (Tween^®^ 80)
Sorbitan fatty acid esters	Sorbitan monolaurate (Span^®^ 20) Sorbitan monostearate (Span^®^ 60) Sorbitan monooleate (Span^®^ 80)
Polyoxyethylene castor oil derivatives	Polyoxyl 35 castor oil (Cremophor^®^ EL)
Polyethylene glycol based derivatives of Vitamin E	d-Alpha-Tocopheryl Polyethylene Glycol-1000 Succinate (TPGS)
Phospholipids, PEG based Phospholipids	Lecithin, Modified Lecithin

**Tab. 3:** Commonly used co-emulsifiers in Isotropic lipid-based formulations.

Classifications	Co-emulsifiers
PEGylated glycerides	PEG-6glyceryl monooleate (Labrafil^®^ M1944 CS)
Monoglycerides of long-chain fatty acids	Glycerol monooleate, Glycerol monostearate
Monoglycerides of medium-chain fatty acids	Glyceryl caprylate/caprate (Capmul^®^ MCM)
Mono and diglycerides of medium-chain fatty acids	Imwitor^®^ 972, Imwitor^®^ 988
Propylene glycol monoester of medium- chain fatty acids	Propylene glycol monocaprylate (Capmul^®^ PG-8; Capryol^®^ 90)
Propylene glycol diester of medium- chain fatty acids	Propylene glycol dicaprylate/dicaprate (Captex^®^ 200)
Poly-glycerol esters	Glyceryl tri-oleate, decaglycerol mono-oleate

**Tab. 4:** Lipid formulations with improved oral bioavailability of API

Compound	Study Design	Formulation	Observation and comments
Phenytoin [31]	BA study in rats after gastric administration	Corn oil suspension, corn oil emulsion	BA and C_max_ from emulsion> corn oil suspension> aqueous suspension
Steroid [32]	Oral BA study in rats	Solution and suspension in sesame oil	BA from lipid solution 3-fold> aqueous suspension, absorption was a function of solubilised drug
Lipophilic pro- drugs of phenytoin [33]	BA study in rats after gastric administration	Solution in short chain fatty acid, TG	BA 4-fold > solution of phenytoin sodium
Antimalarial amine [34]	Oral BA study in beagle dogs	Oleic acid solution	BA from lipid solution 3-fold > standard capsule
Cinnarizine [35]	Oral BA study in beagle dogs	Oleic acid solution	BA from lipid solution 4-fold > standard tablet

BA…bioavailability.

**Tab. 5:** Influence of the digestibility of the lipid vehicle on the rate and extent of drug absorption.

Compound	Study Design	Lipids	Observation and comments
Triamterene [36]	Oral BA study in rats	Peanut oil, oleic acid, triolein	BA from OA > PO > TO aqueous suspension. With 20 μl volumes, PO ≅ aqueous suspension
Penclomedine [37]	BA in rats after intraduodenal administration	SCT/MCT/LCT, mineral oil	BA from MCT > LCT > mineral oil > SCT > aqueous suspension; BA a function of digestibility of vehicle
Cyclosporine [38]	Intraduodenal administration to rats	Lipid micro emulsion	Absorption decreased in bile duct ligated and pancreatomised rats

BA…bioavailability.

**Tab. 6:** Marketed one-lipid excipient formulations

Marketed formulation	API	Dosage form	Manufacturer	Country of sale
Heminevrin^®^	Clomethiazole	Capsule (soft gelatin)	AstraZeneka	UK
Marinol^®^	Dronabinol	Capsule (soft gelatin)	Solvay Pharmaceuticals	USA
Epadel^®^	Ethyl icosapentate	Capsule (soft gelatin)	Mochida Pharmaceuticals	Japan

**Tab. 7:** Marketed two-lipid excipient formulations

Marketed formulation	API	Dosage form	Manufacturer	Country of sale
Alfarol^®^	Alfacalcidol	Capsule (soft gelatin), solution and powder	Chugai Pharmaceuticas	Japan, Taiwan
Hectorol^®^	Doxercalciferol	Capsules (soft gelatin)	Draxis Pharma	USA
Glakay^®^	Menatetrenone	Capsules (soft gelatin)	Eisai Co., Ltd.	Japan

**Tab. 8:** Marketed three-lipid excipient formulations

Marketed formulation	API	Dosage form	Manufacturer	Country of sale
Agenarase^®^	Amprenavir	Capsule (soft gelatin)	GlaxoSmithKline	USA
Targretin^®^	Bexarotene	Capsule (soft gelatin)	Ligand Pharmaceuticals Incorporated	USA
Norvir^®^	Ritonavir	Capsule (soft gelatin)	Abbott Laboratories	USA

**Tab. 9:** Marketed four-lipid excipient formulations

Marketed formulation	API	Dosage form	Manufacturer	Country of sale
Aptivus^®^	Tipranavir	Capsule (soft gelatin)	Boehringer Ingelheim Pharmaceuticals	USA
Lamprene^®^	Clofazimine	Capsule (soft gelatin)	Novartis	Switzerland
Infree^®^	Indomethacin farnesil	Capsule (soft gelatin)	Eisai Co., Ltd	Japan

**Tab. 10:** Marketed lipid-based, extended release formulations.

Marketed formulation	API	Dosage form	Manufacturer	Country of sale
Ketas^®^	Ibudilast	Capsule (hard gelatin)	Kyorin Pharmaceutical Co., Ltd.	Japan
Detrol^®^ LA	Tolterodine Tartarate	Capsule (hard gelatin)	International Processing Corporation	USA
MXL^®^	Morphine Sulphate	Capsule (hard gelatin)	Napp Pharmaceuticals Limited	UK

**Tab. 11:** Marketed, lipid-based microemulsions.

Marketed formulation	API	Dosage form	Manufacturer	Country of sale
Neoral^®^	Cyclosporine A	Capsule (soft gelatin)	Novartis Pharmaceuticals Corporation	USA
Fenogal^®^	Fenofibrate	Capsule (hard gelatin)	SMB Laboratories Novartis	UK
Sandimmune^®^	Cyclosporine A	Capsule (soft gelatin)	Pharmaceuticals Corporation	USA

**Tab. 12:** Marketed, lipid-based oral solutions.

Marketed formulation	Active molecule	Dosage form	Manufacturer	Country of sale
Agenerase^®^	Amprenavir	Oral solution	GlaxoSmithKline	USA
Sustiva^®^	Efavirenz	Oral solution	Bristol-Myers Squibb Company	USA
Rocaltrol^®^	Calcitriol	Oral solution	Roche Laboratories Inc.	USA

**Tab. 13:** Marketed, lipid-based oral suspensions

Marketed formulation	API	Dosage form	Manufacturer	Country of sale
Cipro ^TM^	Ciprofloxacin	Oral suspension	Bayer Healthcare Pharmaceuticals Inc.	USA
